# CT-guided intratumoural administration of cisplatin/epinephrine gel for treatment of malignant liver tumours

**DOI:** 10.1038/sj.bjc.6600116

**Published:** 2002-02-12

**Authors:** T J Vogl, K Engelmann, M G Mack, R Straub, S Zangos, K Eichler, K Hochmuth, E Orenberg

**Affiliations:** Department of Diagnostic and Interventional Radiology, JW Goethe University of Frankfurt, Theodor-Stern-Kai 7, 63590 Frankfurt, Germany; Matrix Pharmaceutical, Inc., 34700 Campus Drive, Fremont, California, CA 94555, USA

**Keywords:** locoregional chemotherapy, interventional radiology, livermetastases of colorectal cancer, hepatocellular carcinoma

## Abstract

To analyze prospectively the interventional and clinical aspects of computed tomography-guided direct intratumoural injection of a novel chemotherapeutic administration and the parenchymal changes of tumour and necrosis in malignant liver tumours. Eight patients with 17 colorectal liver metastases were treated with a mean of 5.1 injections and nine patients with 13 hepatocellular carcinoma nodules with a mean of 3.1 treatments with computed tomography guided local applications of a novel cisplatin/epinephrine gel. This application provides a higher local and lower systemic drug concentration. Volumes of tumour and necrosis prior and after treatment were measured by computer generated volumetric analysis. Contrast enhanced studies verified pretherapeutic viable tumour volumes with a value of 77.4 ml in the metastases and 29.2 ml in the hepatocellular carcinoma nodules. Intratumoural drug application resulted in a significant increase of necrosis and a decrease in viable tumour volume to be 68.3 ml in metastases and 14.5 ml in hepatocellular carcinoma. Local therapy control rate for the follow up to 6 months was 38 and 71% for the group of metastases and hepatocellular carcinoma, respectively. Direct intratumoural injection of cisplatin/epinepthrine injectable gel is a feasible and good tolerated method and results in the development of a statistically significant increase in necrosis in malignant liver tumours. For hepatocellular carcinoma a higher local therapy control rate compared to colorectal metastases can be reported.

*British Journal of Cancer* (2002) **86**, 524–529. DOI: 10.1038/sj/bjc/6600116 www.bjcancer.com

© 2002 Cancer Research UK

## 

Primary and secondary liver malignancies are the most frequent cause of death in oncologic patients, the only potential curative treatment is surgical resection. In patients with colorectal liver metastases, only 10 to 30% are considered for a resection (August *et al*, 1985; Schlag *et al*, 1991); in patients with an isolated liver involvement, 20 to 40% (Lorenz *et al*, 1997). If resection is not possible, then improved quality of life through maximal control of liver lesions must be the goal. Treatment options include systemic or regional chemotherapy, transarterial chemoembolization, percutaneous ethanol injections, laser-induced interstitial thermotherapy, cryotherapy and radio-frequency ablation.

As a palliative treatment with lower side effects regional chemotherapy seems to provide higher local drug concentrations, higher response rates, and less systemic toxicity compared to systemic application (Collins, 1984; Rougier *et al*, 1992; Venook, 1997). Direct intratumoural percutaneous injections deliver even higher drug concentrations to selective sites, are minimally invasive, and effectively produce necrosis (Curley *et al*, 1995). A novel injectable gel has been developed containing cisplatin as the chemotherapeutic and epinephrine as the vasoconstricting agent. This allows high drug concentrations to the treatment site and a slow dispersion to the surrounding tissues, thus reducing systemic exposure. In preclinical studies, conventional chemotherapeutic agents such as cisplatin, fluorouracil, or vinblastine administered intratumourally in the gel formulation enhanced drug retention and increased antitumoural efficacy (Curley *et al*, 1995). Injectable gels have been used to treat various spontaneous tumours in veterinary patients and human malignancies such as basal and squamous cell carcinomas and accessible solid tumours of various histologic types (Miller *et al*, 1997; Burris *et al*, 1998). A recent french study showed development of therapy-induced necrosis after percutaneous intratumoural injections of mitoxantrone in primary and secondary liver malignancies (Farrés *et al*, 1998).

## MATERIALS AND METHODS

This study is part of a multicentre, open-label, phase II trial on the safety and efficacy of percutaneous intratumoural administration of Cisplatin/epinephrine (CDDP/epi) injectable gel in patients with hepatic metastases of colorectal cancer and patients with unresectable primary hepatocellular carcinoma. The investigational agent, CDDP/epi gel (IntraDose® Injectable Gel, Matrix Pharmaceutical, Inc., Fremont, CA, USA) is an injectable, biodegradable, viscous gel composed of cisplatin (CDDP, 4 mg ml^−1^); the vasoconstrictor, epinephrine (epi, 0.1 mg ml^−1^ to a maximum of 1 mg per injection); a protein carrier matrix (purified bovine collagen, 2 mg ml^−1^) as a gellant; and other inactive excipients as buffering and osmotic agents. The components are mixed within 2 h of use. The IntraDose® gel has a gelatinous consistency at room temperature and can flow through a 19.5-gauge needle for direct percutaneous injection in the liver tumour.

### Patients

Seventeen patients were included, nine patients with unresectable hepatocellular carcinoma (HCC) and eight patients with unresectable colorectal liver metastases (CRLM). Seventeen CRLM in eight patients were treated with an average of 5.1 injections per patient (range 1–8) and in nine patients with HCC 13 lesions were treated with an average of 3.1 injections (range 1–4). The number of injections depended on parameters such as tumour size and general condition of the patient. The average patient age was 67.1 years (range 39–79 years), 13 patients were male, four female. All patients had previously undergone other oncologic treatments before entering the study. One patient with HCC had prior chemoembolization for seperate lesion, all patients with colorectal metastases had received systemic chemotherapy with 5-Fluorouracil and folinic acid and three patients had undergone liver resection. All patients had either progressive or recurrent disease after previous treatment.

The study is open to adult patients with up to three intrahepatic biopsy proven tumours (HCC or metastases of colorectal carcinoma) with no extrahepatic spread, Karnofsky Performance Status should be 40 or more and the laboratory results have to be within ranges defined by protocol. Coronary artery disease, history of gastro-oesophageal varices, encephalopathy or bleeding from liver tumours as well as known hypersensitivity to one of the ingredients of the gel or radiographic contrast agents were exclusion criteria. Patients signed a written informed consent proven by the local ethics committee.

### Study design

Prior to treatment patients had a complete history, physical examination, laboratory studies and helical contrast-enhanced computed tomography (CT) scans. Treatment consisted of up to 4 weekly percutaneous intratumoural administrations of IntraDose® gel with up to 10 ml gel (=40 mg Cisplatin) at each treatment session. A second cycle of four treatments could follow the first at the discretion of the investigator. Contrast-enhanced helical CT scans were performed at 2 weeks, 2 months and 6 months after each treatment cycle. After 6 months patients were followed at 3 month intervals by visits or telephone calls.

### Injection technique

**Figure 1:**
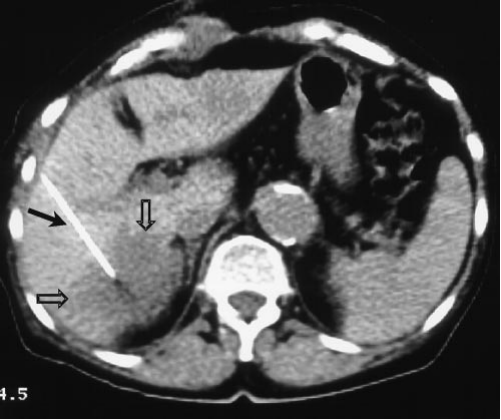
Visualization of the injection procedure in a hepatic metastasis (open arrows) shown in segment 5 using the CARE vision CT programme. Note the needle placed centrally in the tumour (arrow).

Before each treatment, patients are hydrated with intravenous infusions of 500–1000 ml of saline, 0.9% sodium chloride solution. After a plain CT scan the intrahepatic administration of IntraDose® gel was performed under local anaesthesia. To allow a safe lesion localization and homogeneous distribution of the gel throughout the whole tumour volume, several localizations of the needle tip are required in most cases. For this a computer-generated guidance system (CARE® Vision CT, Siemens, Erlangen, Germany) which made nearly online visualization possible was used (see Figure 1

) (Froelich *et al*, 1997). A 19.5-gauge stainless steel puncture needle with six side holes at the tip to support the distribution of the gel was utilized (SOMATEX®, Berlin, Germany). Blood pressure and heart rate were monitored during the treatment procedure. Afterwards the patient had 6 h bed rest.

### Imaging procedures

Hepatic lesions were evaluated via unenhanced and contrast-enhanced CT studies performed with a helical CT scanner (SOMATOM Plus 4®, Siemens AG, Erlangen, Germany). Continuous 5-mm-slice acquisition was used. After the unenhanced CT images were obtained, 120 ml of a non-ionic contrast agent (Ultravist 370®, Schering AG, Berlin, Germany) was administered. The contrast-enhanced helical CT scans were obtained in three phases with the indicated volume, flow, and time delay parameters: arterial phase (volume, 120 ml; flow, 2.5 ml s^−1^; time delay, 20 s); portal venous phase (120 ml, 2.5 ml s^−1^, 70 s); and venous equilibrium phase (120 ml, 2.5 ml s^−1^, 300 s). Image reconstruction parameters included 5-mm slice thickness; 7.5 mm per second feed/rotation, and 5 mm increment.

### Quantitative analysis

Quantitative analyses were performed using the region-of-interest (ROI) technique to determine viable tumour volume and volume of tumour necrosis in the CT scans. The tumour/necrosis areas were marked manually on each CT slice at the CT console, then the volumes were calculated using also the slice thickness. This is one of the most accurate means to determine volumes in CT. Tissues enhanced with contrast media were assumed to be viable and unenhanced tissue nonviable or necrotic. Tumour size, viable tumour volume, and tumour necrosis were assessed before treatment, 2 weeks, 2 and 6 months after treatment then at 3-month intervals.

## RESULTS

**Figure 2:**
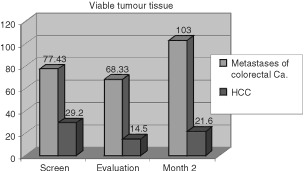
Volumetric measurements of viable tumour volumes before treatment (screen) and 2 weeks and 2 months after last treatment.

Pretherapeutic initial volumetric CT imaging revealed a mean tumour volume per patient of 91.3 ml in colorectal metastases (range 2–125 ml, median 107 ml) and 31.4 ml in HCC (range 1–113 ml, median 16 ml). Measurements of pretherapeutic tumour necrosis in the contrast enhanced studies resulted in a mean value of 13.9 ml for the group of metastases and in 0.4 ml for the group of HCC, the measurements of viable tumour resulted in 77.4 ml for the metastases and 29.2 ml for HCC. After intratumoural drug application quantitative CT evaluation revealed an increase of necrosis in contrast to the initial studies in all patients. Mean necrotic volume 2 weeks after the final treatment resulted in a decrease in viable tumour revealed to be 68.3 ml for metastases (tumour/necrosis volume: 176.4/108.1 ml) and 14.5 ml for HCC (tumour/necrosis volume: 41.8/27.3 ml). However viable tumour at Month 2 control showed a progression again especially for the CRLM to mean 103 ml (tumour/necrosis volume: 219/116 ml) and for the HCC to 21.6 ml (tumour/necrosis volume: 42/ 20.4 ml) (see Figure 2

). Statistical calculations using the Student *t*-test did show that the increase of necrosis after therapy for the HCC is statistically significant (*P*=0.001/0.003 after 2 weeks/2 months with a 95% confidence interval), the change of tumour volume is not significant (*P*=0.65). Although vital tumour in HCC decreased from 29.2 to 14.5 ml this change is not statistically significant (*P*=0.3). In the group of CRLM a significant increase in necrosis (*P*=0.001/0.002 after 2 week/2 months) but also in tumour volume (*P*=0.05) was found. Also as in HCC the decrease in vital tumour was not significant (*P*=0.44 after 2 months).

Evaluable for efficacy were seven patients with colorectal metastases and eight patients with HCC. One patient with colorectal metastasis withdrew from treatment after the first injection, in one patient with HCC and progressive liver cirrhosis treatment was discontinued after one injection due to worsening laboratory values.

The CT scans revealed a variety of tissue changes in the peripheral area of the tumour/necrosis. In colorectal metastases, the hypodense areas were larger in comparison to the initial tumour volume and showed an inhomogeneous contrast enhancement and poorly defined margins. The HCC nodules generally developed a more sharply defined necrosis and no contrast enhancement in the peripheral areas. If the follow up control scans did not show signs of remaining tumour activity, these changes were attributed to oedema and peritumoral reactions. In addition, remaining vessels with a relevant perfusion rate could be detected next to the treated region in the arterial phase of the CT scan.

**Figure 3:**
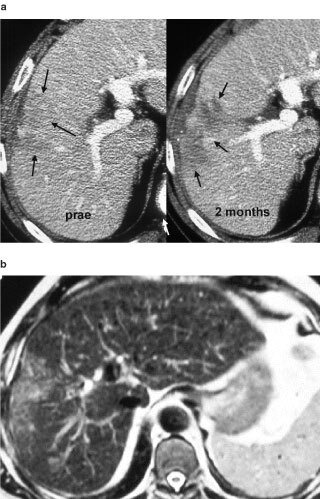
(**a**) 69-year-old patient with histopathologically proven HCC in segment 5/8. Pretreatment CT scan on the left side, lesion identified with decreased attenuation values. CT scan 2 weeks after the fourth/last treatment with IntraDose® gel. Segment 5/8 reveals an irregular area of necrosis that developed after treatment (arrows). Both scans portal venous phase. (**b**) MRI scan (T2-weighted) 2 years after treatment, The lesion is still detectable but has not changed in size, the patient is in a good clinical condition.

The CT-Fluoroscopy supported an easy and safe performance of the injections. Complications due to intravascular or intrabiliary injections, significant bleeding or a pneumothorax were not observed. The modified treatment needle permitted adequate distribution of the gel within the tumour, although a better visualization of the gel would be desirable. Small tumours with a volume of a few ml could be reached without complications. For example, a HCC nodule of initially 3 ml showed complete necrosis after four injections without signs of tumour viability both in the 6 month follow-up CT scan and later studies. In the Figures 3a–c

and 4a,b

**Figure 4:**
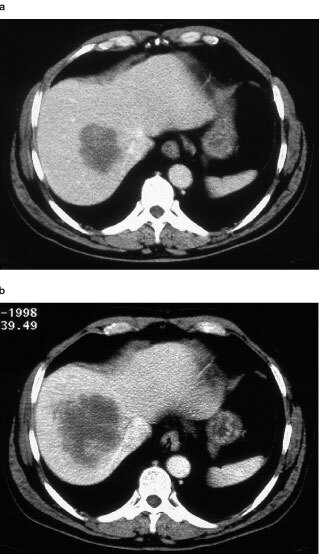
A 55-year-old patient with colorectal metastases to the liver. (**a**) Pretreatment CT scan in segment 8 (portal venous phase). (**b**) CT scan (portal venous phase) at 8 weeks after the pretreatment evaluation and 2 weeks after the fourth treatment. Increased tumour necrosis is evident after treatment.

, examples of treated tumours are shown.

### Clinical follow up

In the patients with liver metastases, CT-evaluation revealed local tumour control in only three patients with a total of four lesions (local tumour control rate 26%). In six patients (75%), new intrahepatic tumours were detected. Local tumour control was defined as complete inactivity of the treated lesions in the 3 and 6 month follow-up scans. Of the eight patients in the HCC group local tumour control rate was 70%. Three patients developed new HCC manifestations (43%). Two patients with worsening liver function and interrupted treatment were not included in the evaluation for local tumour control rate. At this time all eight patients with colorectal metastases and seven of the nine HCC patients died as a result of their disease. During the period for the determination of the control rates only one patient with HCC received another anti-tumour therapy (chemoembolization), no systemic chemotherapy was administered.

The survival rates were calculated with the Kaplan–Meier method. The overall cumulative survival for all 17 patients included was 13.15 months (median 10.17 months; mean 95% Confidence interval 9.25–17.06 months). The survival of the eight patients with CRM was 14.48 months of the nine patients with HCC, 14.11 months. This data can only show a tendency since the number of patients is very small.

### Clinical effects: tolerance, side effects

Overall the treatment procedure was well tolerated. During and immediately after injection the patients suffered from transient local pain (76%), an increase in blood pressure, and heart palpitations during injection (24%) and sweating or shoulder pain (each 30%). Nausea and vomiting were initially seen in 53%, but could be reduced with pretherapeutic infusion of cortisone (Merck, Darmstadt, Germany) and ondansetron (Glaxo Wellcome, Bad Oldesloe, Germany). Clinical signs of cisplatin-induced toxicity like nephrotoxicity, peripheral neurotoxicity or ototoxicity did not occur. Laboratory results were obtained before every treatment and evaluation and did not show major toxicity (for toxicity see Table 1

**Table 1:**
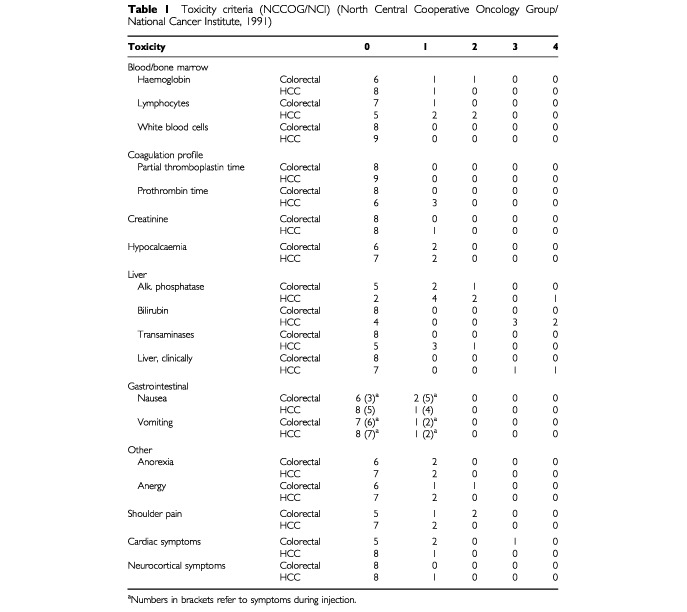
Toxicity criteria (NCCOG/NCI) (North Central Cooperative Oncology Group/National Cancer Institute, 1991)

). Transient elevation of alkaline phosphatase, transaminases and a slight decrease in haemoglobin and lymphocytes could be detected in some patients but never with therapeutic consequences. One patient with hepatitis induced Child-Pugh B classified liver cirrhosis (total bilirubin initially 3.4 mg dl^−1^) died due to liver failure (increasing bilirubin results 10 days after the first treatment). Another patient with Child-Pugh B classified liver cirrhosis (total bilirubin initially 4.1 mg dl^−1^) showed increasing bilirubin values resulting in termination of treatment. Therefore a connection between the worsening liver function and treatment must be considered. The death was assessed as a serious adverse event.

## DISCUSSION

In order to increase the therapeutic index of the drugs, regional treatments for primary and secondary liver tumours are in use. Regional drug infusion parameters, like the hepatic extraction and the regional blood flow, are influencing the pharmacokinetic properties. The collagen matrix gel mixed with cisplatin and epinephrine alters both these factors. It maintains the cisplatin at high concentrations in the treated tumour and so induces an increased cellular extraction of the drug. Further, it reduces the blood flow in the region of the tumour by three factors: the vasoconstriction caused by the epinephrine which is added to the collagen and the cisplatin, the compression of the tumour vessels by the increasing intratumoural pressure following injection and by occlusion of tumour vessels by collagen matrix gel infiltrating into tumour vascular spaces (Davidson *et al*, 1995).

Preclinical studies indicated that via the local application of the IntraDose® gel 20 to 30 times and higher intratumoural cytostatic concentrations can be achieved compared to systemic application.

The antitumourous effect is thought to be due to the combination of substances as epinephrine gel alone did show rapid tumour recurrence in veterinary trials, and addition of epinephrine to the Cisplatin gel showed an improvement of the effect.

Additionally pretherapeutical hydratation of the patient is expected to further reduce systemic toxicity (Hayes *et al*, 1977). Curley *et al* (1995) are reporting on the induction of a significant tumour necrosis and reduction in tumour volume in lesions as large as 12 cm in diameter in human liver malignancies. No cisplatin-related toxicity was reported, the treatments were well tolerated with a transient pain at the injection site and in the liver from stretching the liver capsule (Curley *et al*, 1995). These published data are offering the basis for our *in vivo* studies.

The effects of percutaneous intratumoural administration of Intradose® gel have to be compared with systemic chemotherapy and other local treatments such as local ethanol instillation, chemoembolization and arterial chemo perfusion as well as to no treatment.

The comparative analysis of our data shows that CT-guided intratumoural injection of CDDP/epi gel has induced a statistic significant increase in necrosis in both groups. However, the local effectiveness based on the rate of local recurrences was higher in patients with HCC *vs* the patients with metastases, in HCC local tumour control was 70% and in CRLM only 26%. The response in CRLM is comparable with these after systemic chemotherapy which mainly remains below 30% (Kemeny *et al*, 1993). For the HCC our results are comparable to these after chemoembolization or hepatic arterial infusion, a response of 30–60% after TACE (Takayasu *et al*, 1989; GTCH 1995; Colella *et al*, 1998) and 40–50% after HAI (Carr, 1996; Patt *et al*, 1997) is reported. Here complications due to hepatobiliary toxicity may occur, especially in cirrhotic patients when the liver function is reduced (Ravoet *et al*, 1993; Kemeny, 1995). However complete necrosis of 80–90% can be reached with percutaneous ethanol injection in small HCC (Livraghi *et al*, 1999).

An efficient and innovative treatment for malignant liver tumours – metastases and HCC – in patients with unresectable lesions is MR-guided laser-induced thermotherapy (LITT). This minimally invasive, locoregional technique results in a reliable local tumour control rate of more than 95% in lesions ⩽40 mm in diameter (Vogl *et al*, 1998). Similar to LITT percutaneous radiofrequency (RF) ablation is another ablative method used to induce thermal coagulation of tumour tissue. Both are competitive methods to surgical resection and should not be responded to in detail in this work.

The different results in both groups might be due on the one hand to the different morphology and growth pattern in the HCC nodules. These tumours are regularly encapsulated and have a lower degree of peritumoural spread; thus, the intratumoural pressure after injection may be higher in HCC and may result in a more homogeneous distribution of the drug. On the other hand the group of patients with CRLM had a more palliative character reflected in the high number of patients that developed new intrahepatic metastases.

The survival data has to be judged carefully since the number of patients is relatively low in our study. But an advantage in survival can be seen to emerge in contrast to no treatment, in the CRLM mean survival with 14.6 months is higher than the 7.5 months reported by Stangl *et al* (1994) in a large German study on patients with CRLM receiving no treatment. The survival in HCC is approximately similar than with only palliative treament (Okuda *et al*, 1985).

Further trials are continuing to assess the influence of IntraDose® gel on increased survival of patients with non-resectable liver cancers. A second objective is to establish the optimum tumour size criteria for treatable lesions. Also randomized and combined trials with systemic chemotherapy or chemoembolization are planned.

### Conclusion

In summary, direct intratumoural injection of CDDP/epi injectable gel is a feasible and well tolerated method without major toxicity and results in the development of a relevant necrosis in malignant liver tumours. For HCC, a higher local therapy control rate compared to colorectal metastases was seen.
